# Identification and validation of ferroptosis-related genes and immune infiltration in ischemic cardiomyopathy

**DOI:** 10.3389/fcvm.2023.1078290

**Published:** 2023-02-21

**Authors:** Kai Huang, Kun Mei, Jiahao Duan, Ruting Wang, Chun Yang, Bin Wang, Renjun Gu, Ling Yang

**Affiliations:** ^1^Department of Cardiology, The Third Affiliated Hospital of Soochow University, Changzhou, China; ^2^Department of Cardiothoracic Surgery, The Third Affiliated Hospital of Soochow University, Changzhou, China; ^3^Department of Anesthesiology and Perioperative Medicine, The First Affiliated Hospital of Nanjing Medical University, Nanjing, China; ^4^Nanjing University of Chinese Medicine, Nanjing, China; ^5^School of Chinese Medicine and School of Integrated Chinese and Western Medicine, Nanjing University of Chinese Medicine, Nanjing, China

**Keywords:** ischemic cardiomyopathy, ferroptosis, immune microenvironment, bioinformatics analysis, GEO

## Abstract

**Background:**

Cardiomyocyte death is an important pathophysiological basis for ischemic cardiomyopathy (ICM). Many studies have suggested that ferroptosis is a key link in the development of ICM. We performed bioinformatics analysis and experiment validation to explore the potential ferroptosis-related genes and immune infiltration of ICM.

**Methods:**

We downloaded the datasets of ICM from the Gene Expression Omnibus database and analyzed the ferroptosis-related differentially expressed genes (DEGs). Gene Ontology, Kyoto Encyclopedia of Genes and Genomes pathway enrichment analysis, and protein–protein interaction network were performed to analyze ferroptosis-related DEGs. Gene Set Enrichment Analysis was used to evaluate the gene enrichment signaling pathway of ferroptosis-related genes in ICM. Then, we explored the immune landscape of patients with ICM. Finally, the RNA expression of the top five ferroptosis-related DEGs was validated in blood samples from patients with ICM and healthy controls using qRT-PCR.

**Results:**

Overall, 42 ferroptosis-related DEGs (17 upregulated and 25 downregulated genes) were identified. Functional enrichment analysis indicated several enriched terms related to ferroptosis and the immune pathway. Immunological analysis suggested that the immune microenvironment in patients with ICM is altered. The immune checkpoint-related genes (PDCD1LG2, LAG3, and TIGIT) were overexpressed in ICM. The qRT-PCR results showed that the expression levels of IL6, JUN, STAT3, and ATM in patients with ICM and healthy controls were consistent with the bioinformatics analysis results from the mRNA microarray.

**Conclusion:**

Our study showed significant differences in ferroptosis-related genes and functional pathway between ICM patients and healthy controls. We also provided insight into the landscape of immune cells and the expression of immune checkpoints in patients with ICM. This study provides a new road for future investigation of the pathogenesis and treatment of ICM.

## Introduction

Ischemic cardiomyopathy (ICM) is a common cardiovascular disease caused by chronic myocardial ischemia (1). With the development of percutaneous coronary intervention therapy, the survival rate of patients with acute myocardial infarction (AMI) has improved; however, the incidence of ICM has also increased (2). Currently, the clinical diagnosis of ICM is mainly considered as a left ventricular dysfunction in the presence of severe coronary artery disease, including at least one of the following characteristics: prior revascularization or AMI, >75% stenosis in the left main stem or the left anterior descending artery, and two or more coronary vessels with >75% luminal stenosis (3, 4). Moreover, ICM can easily lead to arrhythmia, embolism, and even heart failure, bringing serious social and economic burden (5). Therefore, understanding the etiology and pathogenesis of ICM is of great significance for guiding clinical treatment and improving patient outcomes.

Ferroptosis is an iron-dependent programmed cell death mode newly discovered in 2012, the mechanism of which is different from those of apoptosis, necrosis, and autophagy (6). The recognition and initiation of ferroptosis rely on the involvement of unique genes, which are characterized by mitochondrial atrophy and increased mitochondrial membrane density, and the accumulation of iron and lipid reactive oxygen species (ROS), (7, 8). More studies have shown that ferroptosis plays an important role in various non-neoplastic diseases. Zhang et al. showed that the administration of ferrostatin-1 (Fer-1; ferroptosis inhibitor) significantly prevented pathological myocardial remodeling, dysfunction, and ultrastructural injury in mice with hypertension by suppressing interleukin-6 (IL6)/STAT3 signaling and activating the xCT/glutathione peroxidase (GPX4) signaling (9). Additionally, Fang et al. observed that feeding mice lacking ferritin H a high-iron diet causes severe cardiac injury and hypertrophic cardiomyopathy (10). In brief, ferroptosis may play an important role in the occurrence and development of ICM.

Recently, increasing studies have revealed that the infiltration of immune cells critically affects myocardial ischemia. Kushnareva et al. found that the expression of PD-L1 and the number of CD3+ cells in endothelial cells increased in the myocardium of patients with ischemic heart failure (11). Besides, several bioinformatic comprehensive analyses have also confirmed the difference in immune infiltrates between patients with coronary artery disease and healthy controls (12, 13). However, the mechanisms underlying immune infiltration in ICM remain poorly understood.

In this study, we conducted a systematic bioinformatics analysis to determine whether and how ferroptosis contributes to the development of ICM. Then, we outlined the immune infiltration landscape in ICM. Moreover, we examined the relationship between ferroptosis and the immune infiltration landscape to gain a better understanding of the potential molecular processes during ICM development.

## Materials and methods

### Data sources

Overall, 259 ferroptosis-related genes were obtained from the FerrDb Database^1^ (14). The gene expression dataset of GSE116250 was downloaded from Gene Expression Omnibus.^2^ GSE116250 is in the GPL16791 platform (Illumina HiSeq 2500), which included 13 ICM and 14 healthy heart tissue samples.

### Ferroptosis-related differentially expressed gene analysis

We transformed the probe into a gene symbol in each dataset based on the platform’s annotation file. The repeatability and discrimination of the ferroptosis-related genes in GSE116250 were verified by principal component analysis (PCA). The ferroptosis-related differentially expressed genes (DEGs) between the ICM and healthy heart control groups were analyzed using the “limma” package in R, with the following cutoffs: adjusted *p* value of <0.05 and log2FC > |0.6|. A heatmap and volcano plot were created using the “heatmap” and “ggplot2” packages of R software.

### Functional enrichment analysis of ferroptosis-related DEGs

To investigate the potential biological functions of ferroptosis-related DEGs between the ICM and healthy control groups, Gene Ontology (GO), (15) and Kyoto Encyclopedia of Genes and Genomes (KEGG), (16) pathway enrichment analysis were conducted using the R packages “clusterProfiler” and “DOSE” (16). In addition, the “clusterProfiler” package was used to perform Gene Set Enrichment Analysis (GSEA), (17) to determine the potential mechanisms of c2 (c2.cp.kegg.v7.5.1.entrez.gmt) in the molecular signature database.

### Correlation and protein–protein interaction analysis of ferroptosis-related DEGs

The correlation between ferroptosis-related DEGs was analyzed using Spearman’s correlation in the “corrplot” package of R. Protein–protein interaction (PPI) analysis of ferroptosis-related DEGs was performed using the STRING database^3^ and Cytoscape (version 3.9.1). A combined score of more than 0.4 was applied to construct the PPI network, and the maximal clique centrality (MCC) algorithm was used to obtain the top five genes in the network. Then, the GeneCards database^4^ was employed to find related genes, proteins, drugs, and diseases to learn more about the hub genes.

### Landscape of immune infiltration and immune checkpoint gene analysis

Here, we downloaded the immunological signature gene sets from the GSEA database.^5^ Then, the Single-gene Gene Set Variation (ssGSEA), (18) was employed to evaluate the infiltration of 24 immune cells between the ICM and healthy control groups using the “GSVA” package. Furthermore, we analyzed the differences in the expression of immune checkpoint genes between the ICM and healthy control groups. Finally, we explored the correlation between the top five hub genes and the immune landscape.

### Patients with ICM and healthy controls

Data from 20 patients with ICM and 20 healthy controls were obtained from the Third Affiliated Hospital of Soochow University between August 2021 and March 2022. The clinical diagnosis of ICM was mainly considered as a left ventricular dysfunction in the presence of severe coronary artery disease. The healthy controls were individuals with uncomplicated hypertension or supraventricular tachycardia without structural cardiac disease. This study was conducted according to the Declaration of Helsinki and was approved by the Medical Ethics Committee of the hospital. Written informed consent was obtained from all participants. Venous blood samples from all patients and controls who participated in the study were collected for subsequent analyses.

### RNA extraction and quantitative real-time PCR

The blood samples from the patients with ICM and healthy controls were processed to isolate peripheral blood mononuclear cells (PBMCs) using Ficoll-Paque PLUS (Cytiva, Shanghai, China). Total RNA was extracted from PBMCs using the RNA Extraction Kit (Omega, Guangzhou, China). Reverse transcription was conducted using the PrimeScript RT Master Mix Kit (Takara, Dalian, China). The mRNA level was assessed using the TB Green Premix Ex Taq Kit (Takara, Dalian, China) following the manufacturer’s instructions. The primers used are shown in Supplementary Table S1. The relative mRNA expression was calculated using the 2 − ΔΔCt method with β-actin normalization.

### Statistical analysis

Student’s *t*-test or the Kruskal–Wallis *H*-test was used to compare continuous variables; the chi-square test or Fisher’s exact test was used to compare categorical variables. Spearman’s rank test or Pearson’s correlation coefficient was used to analyze the associations between hub genes and immune cells and immune checkpoint genes. All statistical analyses were performed using R (version 4.1.3) and Statistical Package for the Social Sciences (version 19.0; IBM Corp., Armonk, NY, United States). Two-tailed values of *p* < 0.05 were used to denote statistical significance.

## Results

### Identification of ferroptosis-related DEGs between ICM and healthy control groups

**Figure 1:**
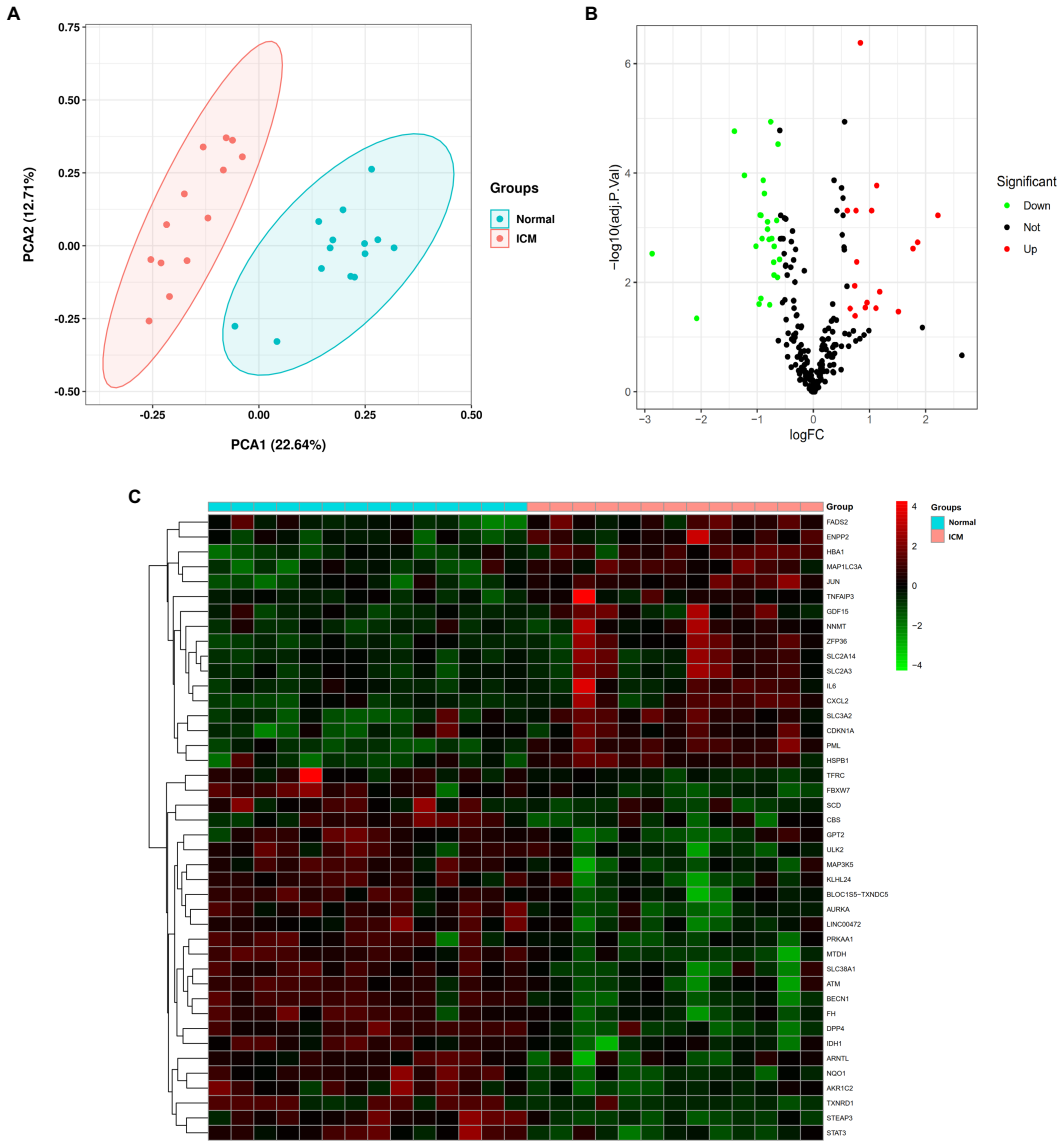
Differentially expressed ferroptosis-related genes in ischemic cardiomyopathy (ICM) and normal samples. **(A)** Principal component analysis for GSE116250. **(B)** Volcano plot of the 259 differentially expressed ferroptosis-related genes. The red dots represent the significantly upregulated genes and the green dots indicate the significantly downregulated genes. **(C)** Heatmap of the 42 differentially expressed autophagy-related genes in ICM and normal samples.

We performed PCA to assess the repeatability and discrimination of 259 ferroptosis-related genes, and the results showed that the repeatability and discrimination of ferroptosis-related genes are fine within the group (Figure 1A). Next, we analyzed the expression of the ferroptosis-related genes in 13 ICM and 14 healthy heart tissue samples, and 42 ferroptosis-related genes were identified using the criteria of an adjusted value of *p* < 0.05 and log2FC > |0.6|. Then, the volcano plot (Figure 1B) and heatmap (Figure 1C) showed the expression pattern of ferroptosis-related DEGs (17 upregulated genes and 25 downregulated genes). The classification of the 42 ferroptosis-related DEGs is shown in Table 1, in which 13 were driver genes, 15 were suppressor genes, and 18 were maker genes. TFRC may be both a driver gene and a marker gene; CBS, HSPB1, and SLC3A2 may be both suppressor and maker genes.

**Table 1:** Classification of 42 ferroptosis-related DEGs.

Maker gene	Driver gene	Suppressor gene
TXNRD1	TFRC	AKR1C2
GPT2	SLC38A1	HSPB1
SLC3A2	BECN1	NQO1
CBS	MAP1LC3A	SLC3A2
KLHL24	ULK2	SCD
GDF15	DPP4	FADS2
BLOC1S5	LINC00472	STAT3
IL6	PRKAA1	PML
CXCL2	TNFAIP3	CDKN1A
TFRC	ATM	ENPP2
HSPB1	MTDH	FH
STEAP3	IDH1	CBS
MAP3K5	FBXW7	ARNTL
SLC2A3		JUN
SLC2A14		ZFP36
HBA1		
NNMT		
AURKA		

### Functional enrichment analysis of ferroptosis-related DEGs

**Figure 2:**
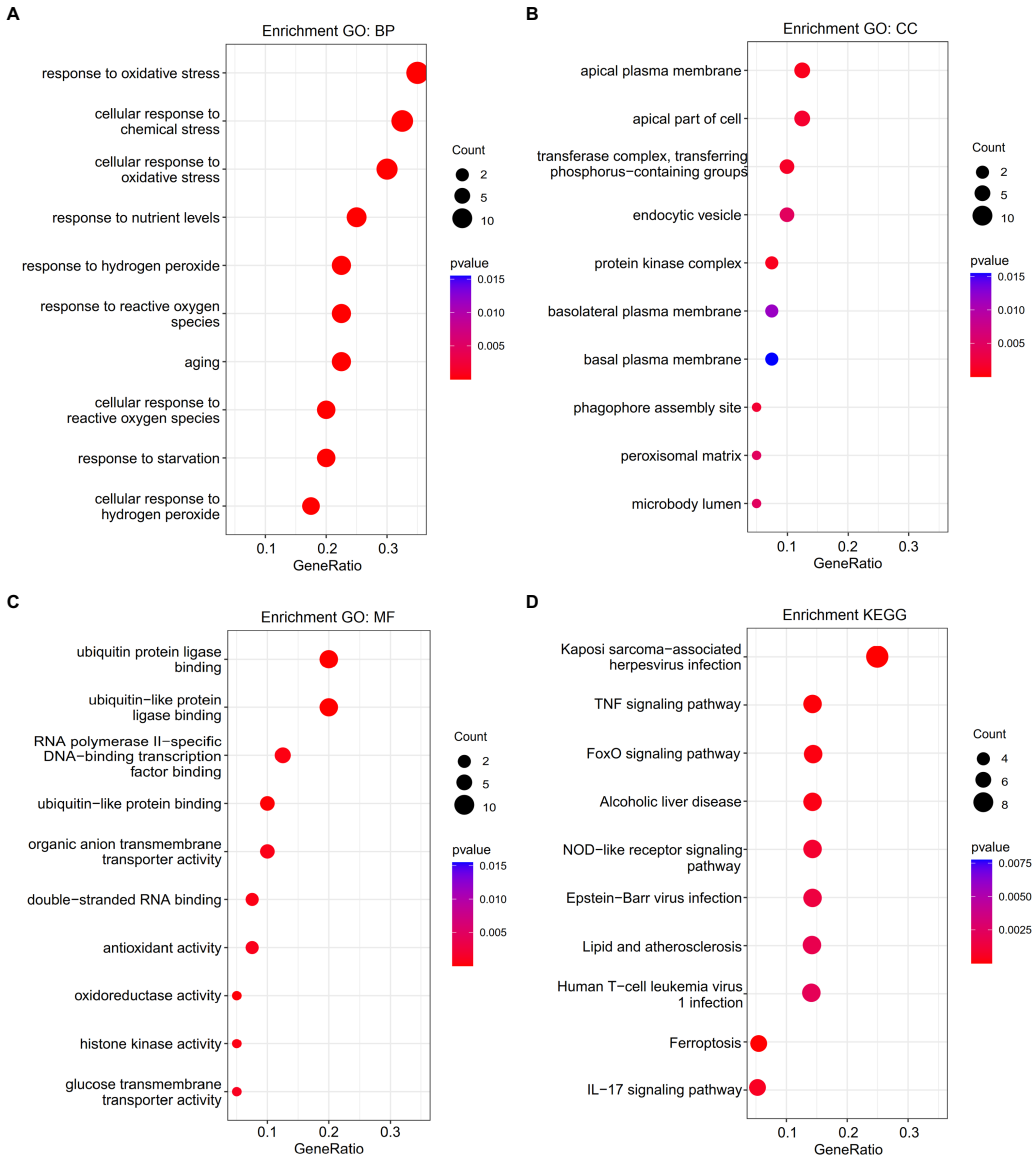
Enrichment analysis of ferroptosis-related differentially expressed genes (DEGs). **(A)** Top 10 GO biological processes pathway. **(B)** Top 10 GO cellular component pathways. **(C)** Top 10 GO molecular function pathway. **(D)** Top 10 KEGG pathway.

**Figure 3:**
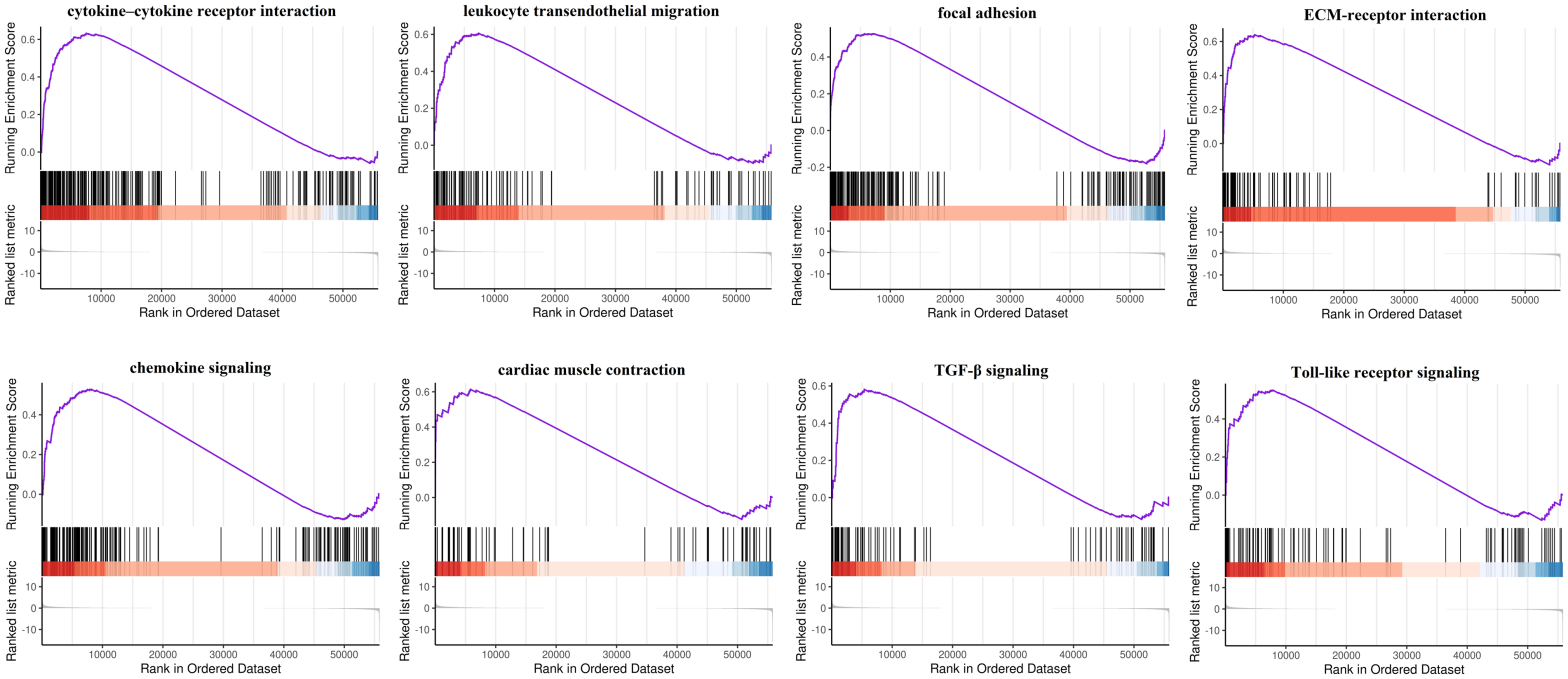
Gene set enrichment analysis (GSEA) of ferroptosis-related genes by the Kyoto Encyclopedia of Genes and Genomes (KEGG) gene set database. FDR < 0.25 and value of *p* < 0.05 were regarded as the cutoff criteria. Most of the ferroptosis-related genes were involved in cytokine–cytokine receptor interaction, leukocyte transendothelial migration, focal adhesion, ECM-receptor interaction, chemokine signaling, cardiac muscle contraction, TGF-β signaling, and Toll-like receptor signaling.

Gene Ontology enrichment was analyzed using the “clusterProfiler” and “DOSE” packages in R (Figures 2A–C). The results of these analyses showed that, regarding the biological process, the genes were mainly enriched in response to oxidative stress and cellular response to chemical stress. As for the cellular component, the genes were mainly enriched in the apical plasma membrane and the protein kinase complex. Finally, regarding molecular function, the genes were mainly enriched in ubiquitin protein ligase binding and ubiquitin-like protein ligase binding. KEGG pathway analysis was performed (Figure 2D). The ferroptosis-related DEGs were mainly focused on the pathways related to ferroptosis, the TNF signaling pathway, and the NOD-like receptor signaling pathway. Furthermore, GSEA also revealed that the expression of the ferroptosis-related genes relevant to cytokine–cytokine receptor interactions, the chemokine signaling pathway, and the Toll-like receptor signaling pathway, among others, were significantly enriched in ICM (Figure 3). The results of the enrichment analysis suggested that oxidative stress, ferroptosis, and immune-related pathways play an important role in ICM.

### Correlation and PPI analysis of ferroptosis-related DEGs

**Figure 4:**
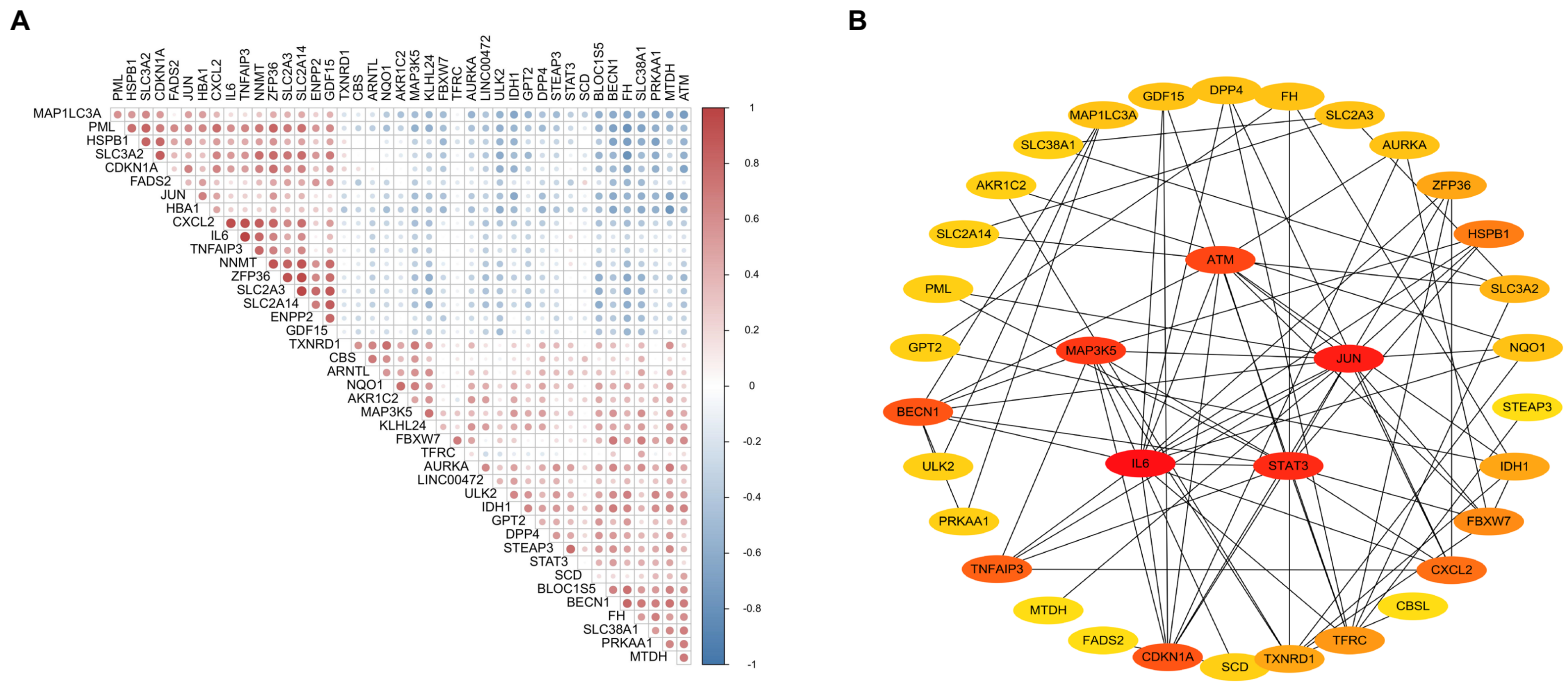
Correlation and protein–protein interaction (PPI) analysis of ferroptosis-related DEGs. **(A)** Spearman correlation analysis of ferroptosis-related DEGs. **(B)** PPI analysis of ferroptosis-related DEGs.

Correlation analysis was performed to investigate the expression correlation of these ferroptosis-related genes. The results showed the relationship among the 42 ferroptosis-related DEGs in the GSE116250 dataset (Figure 4A). According to the combined score of more than 0.4 used to construct the PPI network, we obtained a PPI network (Figure 4B) containing 29 nodes and 82 edges. The MCC algorithm was used to analyze the topological structure of the entire PPI network and score based on the importance of each node. Then, we identified the top five genes (i.e., IL6, JUN, STAT3, MAP3K5, and ATM) as hub genes. Additionally, further details of the selected hub genes are shown through the GeneCards database (Supplementary Table S2).

### Landscape of immune infiltration and immune checkpoint gene analysis between the ICM and healthy control groups

**Figure 5:**
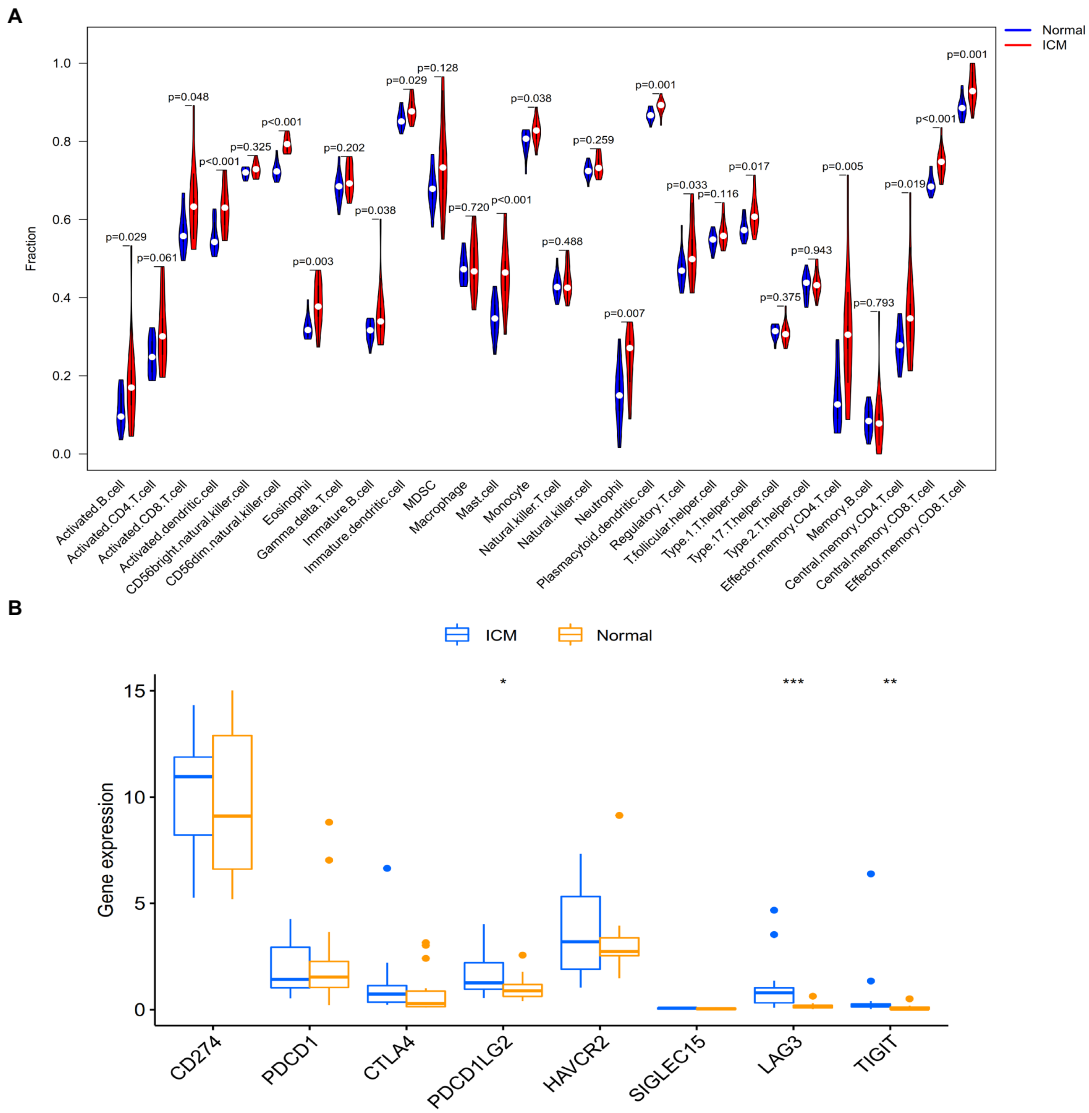
Landscape of immune infiltration and immune checkpoint genes between ICM and normal samples in the GSE116250 dataset. **(A)** Differential expression of different types of immune cells expression between ICM and normal samples. **(B)** Differential expression of different immune checkpoint genes between ICM and normal samples.

In the enrichment analyses, we found that ferroptosis-related DEGs were highly enriched in immune-related pathways. Then, we analyzed the differences in heart tissue immune cells between the ICM and healthy control groups using the ssGSEA algorithm. Among the 24 immune cells, there were 17 cell types with significant differences between the ICM and healthy control tissue samples. As shown in Figure 5A, activated B cells, mast cells, eosinophils, monocytes, neutrophils, type 17 T helper cells, plasmacytoid dendritic cells, and central memory CD8 T cells, among others, had a significantly higher expression in the ICM group. Furthermore, using microarray data, we analyzed the difference in immune checkpoint gene expression between the ICM and healthy control groups. Significant differences in three of the eight immune checkpoint genes were observed. As shown in Figure 5B, PDCDILG2, IAG3, and TIGIT had significantly higher expression levels in the ICM group. In short, the landscape of immune infiltration and immune checkpoint genes was highly activated in ICM.

### Correlation of hub genes with immune cells and immune checkpoint genes

**Figure 6:**
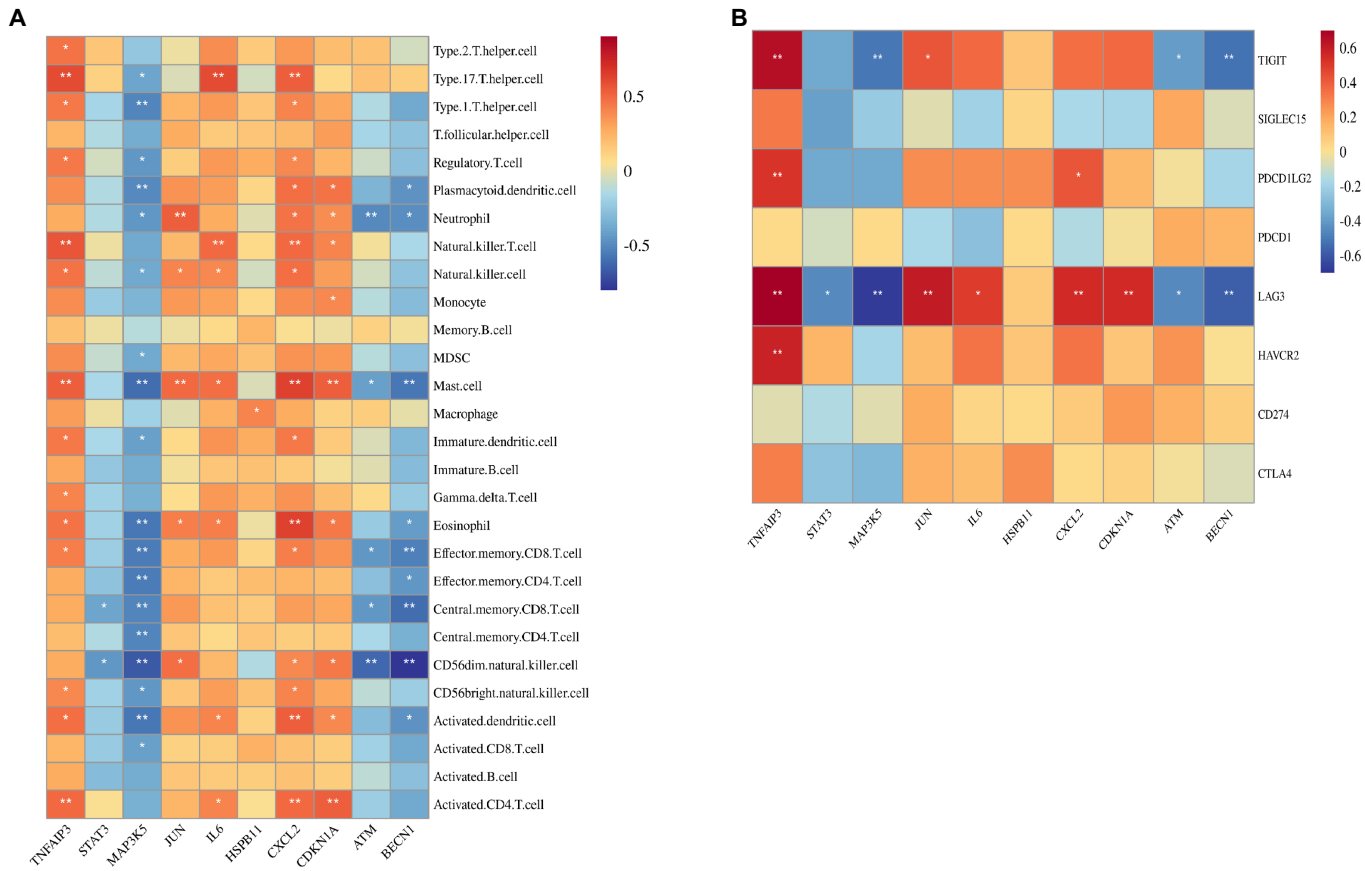
Correlation between hub gene and immune cells and immune checkpoint genes. **(A)** Correlation between hub gene and immune cells. **(B)** Correlation between hub gene and immune checkpoint genes.

Figure 6A shows the correlation between five hub genes and immune cells. IL6 and JUN were positively correlated with the expression of immune cells. IL6 was significantly correlated with six immune cells, among which type 17 T helper cells had the strongest correlation (*r* = 0.60; *p* < 0.001). JUN was significantly correlated with five immune cells, among which neutrophils had the strongest correlation (*r* = 0.536; *p* < 0.01). In contrast, STAT3, MAP3K5, and ATM were negatively correlated with the expression of immune cells. MAP3K5 was significantly correlated with 18 immune cells, among which CD56dim natural killer cells had the strongest correlation (*r* = −0.677; *p* < 0.001). Identically, STAT3 also strongly correlated with CD56dim natural killer cells (*r* = −0.452; *p* < 0.05). ATM was significantly correlated with five immune cells, among which neutrophils had the strongest correlation (*r* = −0.497; *p* < 0.01).

The correlation between the five hub genes and immune checkpoint genes is shown in Figure 6B. IL6 was only significantly associated with LAG3 (*r* = 0.490; *p* < 0.05). JUN was found to be positively correlated with LAG3 (*r* = 0.604; *p* < 0.01) and TIGIT (*r* = 0.425; *p* = 0.05). Moreover, LAG3 was negatively corrected with STAT3 (*r* = −0.456; *p* < 0.05), MAP3K5(*r* = −0.683; *p* < 0.001), and ATM (*r* = −0.458; *p* < 0.05). Therefore, LAG3 may be the most important immune checkpoint gene in ICM, and its expression may be related to the occurrence and development of ICM.

### Verification of the serum concentrations of hub genes in the clinical validation set

**Figure 7:**
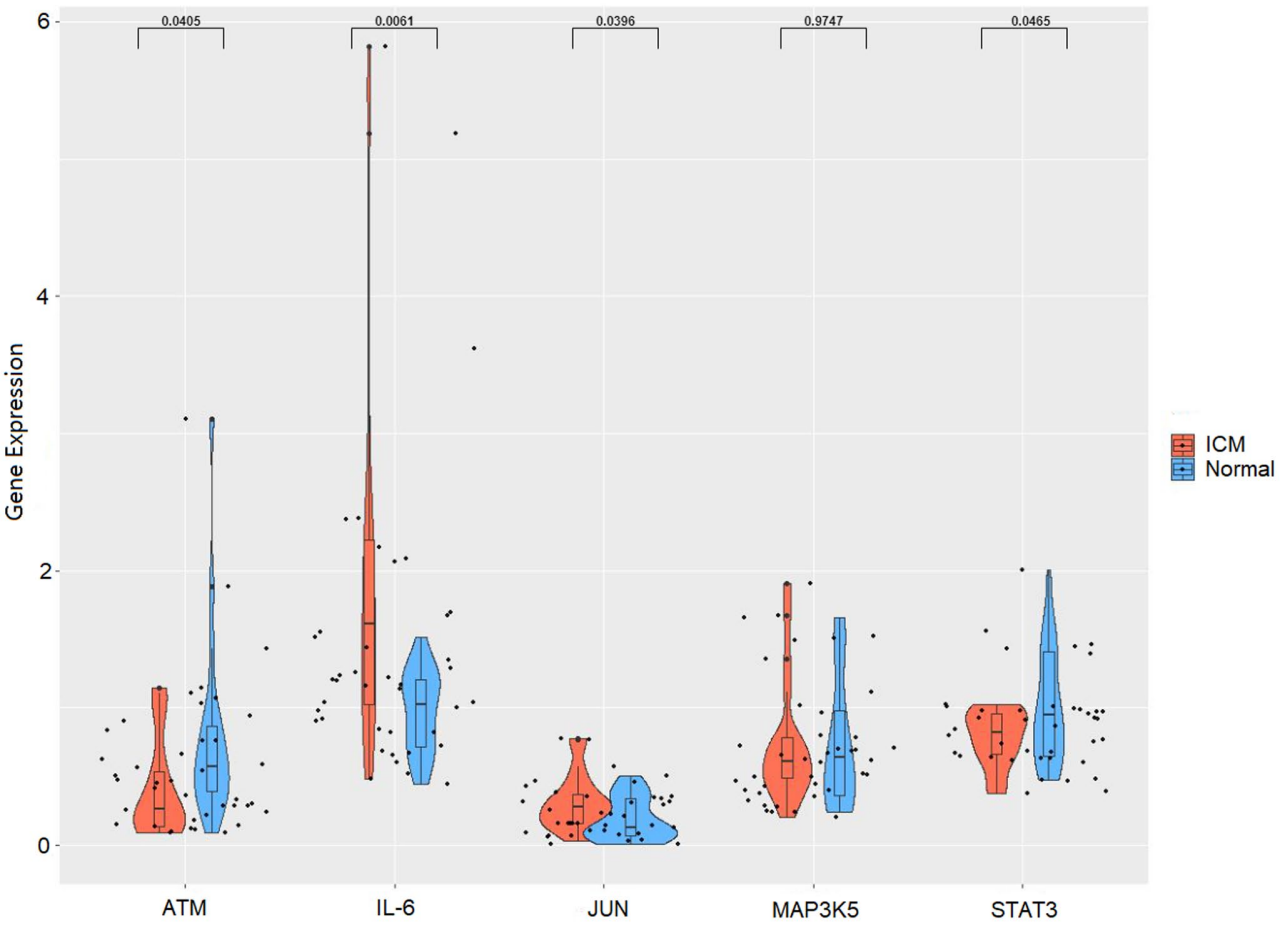
RNA expression of top five hub genes (IL6, JUN, STAT3, MAP3K5, and ATM) were measured in ICM and healthy controls PBMCs samples using qRT-PCR.

To verify the reliability of the analysis based on the GSE116250 dataset, the expression of the top five ferroptosis-related DEGs was further identified using qRT-PCR in our clinical samples. Similar to the results of the mRNA microarray in the heart tissue samples, the expression levels of IL6 and JUN were significantly higher in ICM blood samples, whereas those of STAT3 and ATM were significantly lower (Figure 7).

## Discussion

Ischemic cardiomyopathy is a special type of coronary heart disease (CHD), which refers to long-term myocardial ischemia caused by coronary atherosclerosis, leading to diffuse myocardial fibrosis, cardiac dysfunction, and even heart failure. With the increasing incidence of CHD, the harm caused by ICM to human health is becoming more serious (1, 5). A review summarized that ferroptosis can affect inflammation through immunogenicity and that ferroptosis inhibitors may benefit patients with cardiovascular diseases through their anti-inflammatory effects (19). However, more research is required to enrich our understanding of ferroptosis in the pathogenesis of ICM. This study analyzed different ferroptosis-related DEGs in the ICM and normal groups, and the related functional pathways were preliminarily explored. The imbalance in immune function was inextricably related to the development of ICM. Then, we used the ssGSEA algorithm to analyze the differences in heart tissue immune cells and immune checkpoint gene expression between the ICM and healthy control groups and to analyze the correlation between these cells with hub genes. Finally, we verified the expression of the top five hub genes in PBMCs using qRT-PCR in our clinical samples.

The potential biological functions of these ferroptosis-related DEGs were assessed through GO and KEGG enrichment analyses. These analyses showed that oxidative stress, the TNF signaling pathway, the FoxO signaling pathway, and the NOD-like receptor signaling pathway may play an important role in the ferroptosis process. Studies have shown that oxidative damage caused by oxidative stress was found in the glutamate-induced cell death pathway (20). Meanwhile, glutamate-induced cytotoxicity can be generated by Ca2+ influx or competitive systemXc-(an important component of ferroptosis) after the activation of glutamate receptors, which then mediates the progression of cell ferroptosis (21). Liu et al. revealed that the TNF signaling pathway may be a potential mechanism involved in ferroptosis-related genes in intracerebral hemorrhage (22). Furthermore, another research observed that Fer-1, a ferroptosis inhibitor, can significantly reduce the levels of ACSL4 and TNF-α in mice after MI and then reduce cardiovascular and cerebrovascular injury after recanalization (23). In addition to oxidative stress, the FoxO signaling pathway can promote lipid ROS-mediated cell damage. Kwon et al. found that the metabolic process of polyunsaturated fatty acids can activate the FoxO signaling pathway and then promote the production of ROS (24). The core role of the NOD-like receptor signaling pathway in the innate immune response involves the signal transduction ATP enzyme, which affects atherosclerosis and ICM formation by modulating inflammation that can cause autoimmunity (25, 26). Wu et al. revealed that the excessive activation of NOD-like receptor protein 3 can reduce the activity of GPX4, reduce the antioxidant capacity of cells, increase lipid peroxidation, increase lipid ROS, and cause ferroptosis (27). Anyway, many signal pathways are involved in the ferroptosis process, which directly or indirectly affect the balance of the production and degradation of lipid ROS in cells.

Immune response activation is involved in the occurrence and development of many cardiovascular diseases, regardless of whether they involve innate or adaptive immunity. Through the ssGSEA algorithm, we found that immune cells, including activated B cells, mast cells, eosinophils, monocytes, neutrophils, and type 17 T helper cells, were significantly upregulated in ICM. Li et al. found that ferroptosis can regulate the recruitment of neutrophils to the injured myocardium and that inhibiting ferroptosis can reduce the MI area, improve ventricular systolic function, and reduce adverse ventricular remodeling (28). Dick et al. found that the depletion of intrinsic cardiac macrophages in the early stage of MI would impair cardiac function (29). However, monocyte-derived macrophages are involved in adverse ventricular remodeling after MI, and the level of monocytes in the blood is a prognostic marker of ICM (30). Moreover, a recent study found that CD8 + T cells were recruited and activated in ischemic myocardial tissue after MI in mice, leading to apoptosis of cardiomyocytes (31). Moreover, our analysis showed that multiple immune checkpoints (i.e., PDCDILG2, LAG3, and TIGIT) were also highly expressed in ICM. These overexpressed immune checkpoint factors may affect the immune response to cardiac damage, leading to increased inflammation, decreased collagen deposition, and ventricular remodeling (32, 33).

We obtained five ferroptosis-related DEGs as hub genes based on the PPI analysis, and the expression levels of IL6, JUN, STAT3, and ATM remained different by qRT-PCR validation. Studies have shown that in patients with ICM, high IL6 levels are associated with a poor prognosis (34, 35). Other relevant animal experiments also support this result (9, 36). Li et al. showed that the levels of JUN were significantly higher in patients with CHD and ICM than in healthy individuals by bioinformatics analysis (37). STAT3 is a transcription factor that plays a critical role in heart development and protection. D’Ascenzo et al. found that the inhibition of STAT3 phosphorylation attenuated ischemia–reperfusion injury during interventional therapy for acute coronary syndrome (38). Additionally, in Chinese Han populations, the ATM rs189037 polymorphism is associated with coronary artery disease (CAD), and the rs189037 genotype seems to be associated with a lower risk of CAD and is a protective genetic marker of CAD, particularly in males and smokers (39).

Inevitably, this study also has some limitations. First, although we used the PBMCs of subjects in our center to verify the expression of the hub genes, there was a lack of signal pathway-related mechanisms for further verification. Second, the proportion of immune cells was extrapolated using the ssGSEA algorithm rather than true tests of the number in heart tissue.

## Conclusion

Our study showed significant differences in ferroptosis-related genes between patients with ICM and healthy controls, and oxidative stress and the TNF signaling pathway, among others, may play an important role in the ferroptosis process. We also provided insight into the landscape of immune cells and the expression of immune checkpoints in patients with ICM. However, future studies of ferroptosis in the occurrence and development of ICM need to be further meticulously explored.

## Data availability statement

The datasets presented in this study can be found in online repositories. The names of the repository/repositories and accession number(s) can be found in the article/Supplementary material.

## Ethics statement

The studies involving human participants were reviewed and approved by Ethics Committee of Changzhou First People’s Hospital, Soochow University. The Ethics Committee waived the requirement of written informed consent for participation.

## Author contributions

KH, KM, JD, RW, CY, BW, RG, and LY made a significant contribution to the work reported, whether that is in the conception, study design, execution, acquisition of data, analysis and interpretation, or in all these areas, took part in drafting, revising, or critically reviewing the article, gave final approval of the version to be published, have agreed on the journal to which the article has been submitted, and agree to be accountable for all aspects of the work. All authors contributed to the article and approved the submitted version.

## Funding

This study was supported by grants from the National Natural Science Foundation of China (82070405), Young Talent Development plan of Changzhou Health Commission (CZQM2020004), Basic Research Project of Changzhou science and Technology Bureau (CJ20200104), and Social Development Projects of Changzhou Science and Technology Bureau (CE20205039).

## Conflict of interest

The authors declare that the research was conducted in the absence of any commercial or financial relationships that could be construed as a potential conflict of interest.

## Publisher’s note

All claims expressed in this article are solely those of the authors and do not necessarily represent those of their affiliated organizations, or those of the publisher, the editors and the reviewers. Any product that may be evaluated in this article, or claim that may be made by its manufacturer, is not guaranteed or endorsed by the publisher.

## Footnotes

^1^ http://www.zhounan.org/ferrdb/

^2^ http://www.ncbi.nlm.nih.gov/geo/

^3^ https://cn.string-db.org/

^4^ http://www.genecards.org/

^5^ http://software.broadinstitute.org/gsea/index.jsp
