# Supplementary material for: Identification and validation of ferroptosis-related genes and immune infiltration in ischemic cardiomyopathy
Source: Front Cardiovasc Med. 2023 Feb 21;10:1078290. doi: 10.3389/fcvm.2023.1078290 (PMC9989975; doi:10.3389/fcvm.2023.1078290)
Supplement: Supplementary file 1 [file Table_1.DOCX]

Supplementary Material

**Table S1 Primer sequences used for qRT-PCR.**

| Primer | 5’-3’ |
| --- | --- |
| *IL6*-qPCR-F | CACTGGTCTTTTGGAGTTTGAG |
| *IL6*-qPCR-R | GGACTTTTGTACTCATCTGCAC |
| *JUN*-qPCR-F | CAAACCTCAGCAACTTCAACC |
| *JUN*-qPCR-R | CTGGGACTCCATGTCGATG |
| *STAT3*-qPCR-F | TCGGCTAGAAAACTGGATAACG |
| *STAT3-*qPCR-R | TGCAACTCCTCCAGTTTCTTAA |
| *MAP3K5*-qPCR-F | GGAGAAAGAGATGTCAAGGGAA |
| *MAP3K5*-qPCR-R | CAATTTTGTCTTGGTCTTCCGT |
| *ATM*-qPCR-F | CTGACAATCATCACCAAGTTCG |
| *ATM*-qPCR-R | CTTCCTGAGCTTTCAAGTATGC |

**Table S2 The top five hub genes in ICM.**

| **Gene** | **Genecards Indentifier** | **Full Name** | **Gene-related Diseases** |
| --- | --- | --- | --- |
| IL6 | GC07P022725 | Interleukin 6 | Arteriovenous Malformations of the Brain, Type 2 Diabetes Mellitus, Cardiovascular System Disease, Fatty Liver Disease, Sleep Apnea, Kidney Disease |
| JUN | GC01M058780 | Jun Proto-Oncogene | Ocular Hypertension, Hyperglycemia, Combined Immunodeficiency, Vascular Disease, Cardiomyopathy, Dilated Cardiomyopathy |
| STAT3 | GC17M042313 | Signal Transducer And Activator Of Transcription 3 | Hypereosinophilic Syndrome, Diabetes Mellitus, Myocarditis, Heart Failure, Hypothyroidism |
| MAP3K5 | GC06M136557 | Mitogen-Activated Protein Kinase Kinase Kinase 5 | Non-Alcoholic Fatty Liver Disease, Fatty Liver Disease, Diabetes Mellitus, Alzheimer Disease, Liver Failure, Amyotrophic Lateral Sclerosis 1 |
| ATM | GC11P108222 | ATM Serine/Threonine Kinase | Immune Deficiency Disease, Cerebellar Disease, Acute Anterolateral Myocardial Infarction, Peripheral Vascular Disease, Type 2 Diabetes Mellitus |
